# Prevalence and reasons for using cannabidiol, delta-8 tetrahydrocannabinol, cannabinol, cannabigerol, and hexahydrocannabinol among US adults

**DOI:** 10.1186/s42238-025-00359-8

**Published:** 2025-12-09

**Authors:** Nora Satybaldiyeva, Kevin H. Yang, Wayne Kepner, Karen Ferran, Eric C. Leas

**Affiliations:** 1 Stanford Prevention Research Center, Stanford University School of Medicine, Palo Alto, CA USA; 2 Department of Psychiatry, University of California San Diego School of Medicine, San Diego, CA USA; 3 Stanford University School of Medicine, Palo Alto, CA USA; 4 School of Public Health, San Diego State University, San Diego, CA USA; 5 Herbert Wertheim School of Public Health and Human Longevity Science, University of California San Diego, 9500 Gilman Drive, Mail Code: 0725, La Jolla, CA 94304-1334 USA

**Keywords:** Cannabinoids, Cannabidiol, Delta-8-tetrahydrocannabinol, Cannabinol, Cannabigerol, Hexahydrocannabinol

## Abstract

**Background:**

Since the passage of the 2018 US Farm Bill there has been a market for cannabinoid products derived from *Cannabis sativa L.* that contain < 0.3% delta-9 tetrahydrocannabinol (THC). Understanding the characteristics and motivations of cannabinoid product users is crucial for appropriate regulation of these products.

**Methods:**

We conducted a cross-sectional survey of 1,523 adults 18 years or older using the probability-based Ipsos KnowledgePanel, representative of 97% of US households. We assessed lifetime use of cannabidiol (CBD), delta-8 THC, cannabinol (CBN), cannabigerol (CBG), and hexahydrocannabinol (HHC), as well as self-reported reasons for using these products (i.e., medical vs. recreational). Using multivariable logistic regression models, we investigated associations of demographic and health behavior characteristics with product use. Lastly, we used the Medical Dictionary for Regulatory Activities to code medical reasons for cannabinoid product use into system organ class and preferred term categories.

**Results:**

Lifetime use of CBD was 35.2% (95% CI 32.7–37.9), compared with 7.7% (95% CI 6.5–9.1) for delta-8 THC, 4.5% (95% CI 3.7–5.6) for CBN, 1.3% (95% CI 0.9–1.9) for CBG, and 1.5% (95% CI 1.0-2.1) for HHC. More adults used CBD for medical purposes (71.9%, 95% CI 68.9–74.7) than recreation (47.1%, 95% CI 43.9–50.3), which was also the case for CBN, CBG and HHC. Conversely, more adults used delta-8 THC for recreation (76.1% 95% CI 67.0-83.3) than for medical reasons (50.9; 95% CI 42.6–59.2). The most cited preferred terms for CBD use were anxiety (14.7%, 95% CI 13.0-16.6), pain (13.1%, 95% CI 11.5–15.0) and arthralgia (11.2%, 95% CI 9.5–13.2), for delta-8 THC use they were anxiety (18.6%, 95% CI 13.3–25.3), pain (15.2%, 95% CI 11.1–20.5) and insomnia (10.7%, 95% CI 7.4–15.3), and for CBN use they were insomnia (15.4%, 95% CI 9.6–23.9), pain (11.1%, 95% CI 6.4–18.7) and anxiety (10.9%, 95% CI 6.0–19.0).

**Conclusions:**

Use of cannabinoid products is appreciable, particularly CBD and delta-8 THC. Most adults use CBD, CBN, CBG, and HHC for medical reasons, but delta-8 THC for recreation. Pain, anxiety, insomnia and arthralgia were common medical reasons for use across the different cannabinoids assessed.

**Supplementary Information:**

The online version contains supplementary material available at 10.1186/s42238-025-00359-8.

## Introduction

The passage of the 2018 United States (US) Farm Bill, which separated “hemp” from the definition of “marijuana”, resulted in the growth of a market for cannabinoid products derived from hemp [1]. Derived cannabinoid products are those derived from *Cannabis Sativa L.* that contain less than 0.3% delta-9 THC, which is the main psychoactive compound in cannabis. Some cannabinoids, including cannabidiol (CBD) and cannabigerol (CBG), are naturally abundant in hemp and are typically extracted directly from the hemp plant [2]. However, other cannabinoid products, including delta-8 tetrahydrocannabinol (THC) and hexahydrocannabinol (HHC), are naturally present in the hemp plant in minimal amounts and are therefore often produced via chemical synthesis [3]. The most well-researched and prevalent cannabinoid products are CBD and delta-8 THC [4, 5]. However, there are numerous others, many of which are psychoactive, that are widely available to consumers throughout the US.

The new and unregulated marketplace of cannabinoid products poses several health risks to consumers. First, in US states where cannabis use remains restricted, cannabinoid products, such as delta-8 THC, may be used as substitutes for individuals to achieve similar euphoric effects (i.e., to get “high”) [5, 6]. Cannabinoid products may not be a safe substitution for cannabis as they are not tested for their safety and may contain adulterants resulting from their synthesis. Already, there have been over 2,000 reports of adverse events linked to delta-8 THC consumption received by the Food and Drug Administration (FDA) and the National Poison Control Centers between December 2020 and February 2022, many of which required medical intervention and involved pediatric patients [7–9]. Unintentional youth exposure to cannabinoid products may be a result of their marketing, which mimics popular candy and chip brands familiar to children [7]. Furthermore, many cannabinoid products, such as CBD, might be used to relieve medical symptoms, even though they have not been approved for therapeutic applications [10, 11]. Therefore, it is critical to understand the prevalence and motivations behind derived cannabinoid product use.

There is a scarcity of data to answer basic epidemiological questions about cannabinoid products such as who has used them and why? Examining the reasons for cannabinoid use is particularly important as cannabinoid products can be psychotropic (e.g., HHC) or non-psychotropic (e.g., CBD), which can influence consumer intentions, behaviors, and potential health outcomes. Most of the scientific literature on derived cannabinoid products has focused on CBD and delta-8 THC and relies on online surveys that use convenience samples [12–14]. Assessment of online communities has shown that people may be using CBD for self-treatment of medical symptoms [10]. Although CBD has been FDA approved only for the treatment of epilepsy, in the form of EPIDIOLEX^®^, retailers that sell CBD have been shown to make unsubstantiated health claims related to its properties [15, 16]. While some of these claims are framed as structure/function claims under dietary supplement labeling, it is important to note that the FDA does not officially recognize CBD as a dietary supplement or dietary ingredient. Reasons for using delta-8 THC have not been extensively assessed, with several convenience sample surveys showing that most adults use it for reasons similar to delta-9 THC [12, 13].

Nevertheless, there are very few studies about cannabinoid products other than CBD and delta-8 THC, such as cannabinol (CBN), a commercially available cannabinoid that has some limited scientific literature about its use [17, 18]. A systematic review of pre-clinical research has shown that CBN affects non-cannabinoid receptors that are involved in pain, inflammation, and mood regulation [17]. However, a systematic search of clinical and pre-clinical studies found insufficient evidence to support claims that CBN supports sleep health [18]. One recent study found that CBN was detected in 8.9% of saliva samples among a sample of nightclub attendees in New York City [19].

To date, there is only one nationally representative study among US adults that has assessed past-year derived cannabinoid product use and estimated the prevalence of past-year CBD, delta-8 THC, CBN, and CBG use to be 21.1%, 11.9%, 5.2%, and 4.4%, respectively [20]. However, it did not explore the motivations for use and did not include hexahydrocannabinol (HHC), which is becoming increasingly widespread and has already been linked to adverse events (e.g., insomnia, psychosis) [21]. A national study of 12th grade students found that delta-8 THC may also be commonly used by youth, with 11.4% of adolescents having used delta-8 THC in their lifetime [22]. Similar to the first national survey, this study did not examine motivations for delta-8 THC use and did not assess any other derived cannabinoid products. Therefore, there is a gap in the literature regarding the reasons for using various derived cannabinoid products, which is especially important if individuals are using them as a substitute for the treatment of medical conditions.

Herein, we estimate the lifetime use of derived cannabinoid products using a nationally representative sample of US adults. Furthermore, we identify the characteristics of individuals who use cannabinoid products and their reasons for using these products. Among adults who use derived products for a medical purpose, we categorize and compare their reasons through the use of a standardized medical dictionary. The results of this study may be used by policymakers to make better-informed decisions about their regulation, and public health officials to develop better education campaigns for consumers.

## Methods

### Study sample

The study sample consisted of participants in a US survey on CBD use conducted between October and November of 2023. Respondents were recruited from Ipsos KnowledgePanel^®^, the largest probability-based online panel in the US. The Ipsos KnowledgePanel^®^ consists of about 55,000 panelists whose demographics are weighted to the US census. To recruit panel members, Ipsos uses probability selection algorithms for random-digit telephone dialing and address-based sampling methods. Households without computer and/or internet access are provided with a web-enabled device (e.g., laptop) and free monthly internet access. The KnowledgePanel^®^ is representative of 97% of the adult US general population, covering all 50 states and Washington DC, and has been widely used to provide representative statistics on drug use in the US [23–27].

The KnowledgePanel^®^ recruited a random sample of 4,505 adults aged 18 years or older to complete the survey, with a survey completion rate of 63.9%. A predetermined target sample was specified: 1000 adults who have ever used CBD and 500 adults who have never used CBD, with sample sizes determined to provide representative estimates of CBD use behaviors with < ± 3% margin of error. There were 1,523 adults who qualified for the survey after answering questions about their use of CBD. The final analytic sample consisted of 1008 adults who have ever used CBD and 515 adults who have never used CBD. The survey was administered in both English and Spanish and the average completion time for the survey was 10 min. The survey was considered exempt human subjects research by the Human Research Protections Program at the University of California, San Diego.

### Measures

*Derived Cannabinoid Product Use.* Participants were asked about their familiarity with different cannabinoid products using four separate questions that asked, ‘Before today, have you ever heard of 1) delta-8 THC, 2) cannabinol (CBN), 3) cannabigerol (CBG), and 4) hexahydrocannabinol (HHC)?’ with response options “Yes/No/Don’t know”. Awareness of cannabidiol (CBD) was not assessed since the sampling method of the survey oversampled CBD users. Those who reported hearing about a cannabinoid product were then asked ‘Have you ever, even once, used 1) CBD, 2) delta-8 THC, 3) CBN, 4) CBG, and 5) HHC?’ with response options “Yes/No/Don’t know”. Participants could also refuse to answer any of the survey questions. Those who refused to answer questions about cannabinoid product use or responded “Don’t know” were categorized as having never used the product.

*Reasons for Derived Cannabinoid Product Use.* Participants who reported ever using a cannabinoid product, were asked “Which purposes have you ever used 1) CBD, 2) delta-8 THC, 3) CBN, 4) CBG, and 5) HHC products for?” with response options (1 = I have ONLY used the products for medical purposes; 2 = I have ONLY used the products for recreation; 3 = I have used the products for BOTH medical purposes and recreation; 4 = Don’t know). Medical purposes were defined as “to treat or decrease symptoms of a health condition” and recreation was defined as “to get pleasure, feel good, or relax but not to treat a specific condition”. Individuals who reported using a cannabinoid product for medical reasons were asked “What health condition(s) have you taken 1) CBD, 2) delta-8 THC, 3) CBN, 4) CBG, and 5) HHC for?” with the option to input up to 10 different conditions using an open text field.

*Socio-demographics.* Information was provided by the KnowledgePanel about the sociodemographic characteristics of each respondent.

*Age.* Age was provided as a categorical variable with four levels: (1) 18–29, (2) 30–44, (3) 45–59, and (4) 60 + years.

*Sex.* Sex was provided as a dichotomous variable (1) male or (2) female.

*Race/ethnicity.* Race/ethnicity was provided as a categorical, five-level variable: (1) White, non-Hispanic, (2) Black, non-Hispanic, (3) Other, non-Hispanic, (4) Hispanic and (5) 2 + races, non-Hispanic. For the purposes of this analysis, the Other, non-Hispanic and 2 + races, non-Hispanic were combined.

*Education.* Education level was provided as a categorical, four-level variable: (1) No high school diploma or GED, (2) High school graduate (high school diploma or the equivalent GED), (3) Some college or Associate degree, and (4) Bachelor’s degree or higher.

*Self-reported physical health*,* mental health*,* and quality of life.* The survey asked each participant to self-rate their physical and mental health and quality of life on a 5-point Likert scale ranging from “1) Poor” to “5) Excellent”. As previously done by other studies using this question [28, 29], we categorized all three variables by grouping the “Fair/Poor”, “Good”, and “Very Good/Excellent” categories.

*Cannabis Use.* The survey asked each participant about their use of other substances. Cannabis use was assessed using the question “In your entire lifetime, have you ever used cannabis (i.e., marijuana) that contained THC?” and collected as a dichotomous variable (yes/no).

*Other Drug Use.* Other drug use was assessed using the question “In your entire lifetime, have you ever used any of the following substances?” with response options (1) Cocaine or crack, (2) Non-prescription stimulants like methamphetamine or speed, (3) Psychedelics like LSD or psilocybin-containing mushrooms, (4) Empathogens like, MDMA also known as ecstasy or molly, (5) Dissociative substances like ketamine, (6) Any other drugs like heroin, inhalants, or solvents, and (7) None of the categories apply. Endorsement of any category 1–6 use was coded as “other drug use.”

*Medical Reasons Coding.* The health conditions reported by individuals who used cannabinoid products for medical reasons were coded using a standardized medical language, the Medical Dictionary for Regulatory Activities (MedDRA) version 26.1 [30]. MedDRA uses a five-level hierarchical structure with the “System Organ Class” (SOC) at the top. SOC represents terms based on an anatomical or physiological system, etiology, or function. Three levels beneath the SOC is the “Preferred Term” (PT), which represents a distinct medical concept related to a symptom, sign, or disease diagnosis. The lowest tier in the hierarchy is the “Lowest-Level Term” (LLT), which captures how observations are described in the everyday language by a consumer. All LLTs are associated with a single PT and all PTs are associated with a single SOC.

To translate medical reasons for using cannabinoids to MedDRA, three annotators (N.S., K.H.Y., and W.K.) assigned all medical reasons to MedDRA LLTs. There was no restriction on the number of medical reasons or LLTs a cannabinoid product could be used for. For example, the medical reason “arthritis and asthma,” would be mapped to the LLTs “Arthritis” and “Asthma”. Annotators had moderate agreement on LLT selections (Cohen’s K = 0.718), with differences primarily resulting from lexical variations allowed in MedDRA (e.g., MedDRA has distinct LLTs for “difficulty sleeping” and “poor sleep”). Coder agreement was higher at the PT (Cohen’s K = 0.78) and SOC (Cohen’s K = 0.84) levels; therefore analyses were performed at these levels.

### Statistical analyses

To produce population-based estimates, we used survey weights, which accounted for the oversampling of CBD users in the study design. All percentages were weighted by population parameters based on the findings of the most recent US Current Population Survey. A survey-specific poststratification adjustment was used to account for survey non-response, as well as non-coverage or under-sampling and oversampling resulting from the survey-specific sampling design. All characteristics were categorical and were thus summarized using counts and proportions. Weighted frequencies and the corresponding 95% confidence intervals (CIs) of cannabinoid product use were calculated to determine the proportion for each of the cannabinoid products assessed. Differences in frequency distributions for each outcome and the sociodemographic, substance use, and self-reported health correlates were examined by cannabinoid product use.

Among people who endorsed lifetime use of a cannabinoid product, we calculated weighted frequencies and corresponding 95% CIs for the proportion who used the product for medical and recreational purposes. If a respondent reported using a cannabinoid product for both medical reasons and recreation, they were included in the proportion for both categories.

Multivariable analyses were conducted for outcomes with at least 50 participants endorsing lifetime use of that cannabinoid product, following the common data suppression threshold used by the American Community Survey [31]. For each sociodemographic characteristic, the cannabinoid product used (CBD, delta-8 THC, CBN) was treated as the outcome and a multivariable log-binomial regression model was used to calculate adjusted risk ratios (aRRs) and corresponding 95% CIs adjusting for all other sociodemographic, substance use, and self-reported health factors. All multivariable analyses used complete case analysis. All analyses were performed using R, version 4.1 (R Project for Statistical Computing).

## Results

In this sample of US adults, over a third (35.2%; 95% CI 32.7–37.9) reported ever using CBD (Table 1). Furthermore, 7.7% (95% CI 6.5–9.1) reported ever using delta-8 THC compared with 4.5% (95% CI 3.7–5.6), 1.3% (95% CI 0.9–1.9), and 1.5% (95% CI 1.0–2.1) for CBN, CBG, and HHC respectively.

**Table 1:** Demographic and behavioral characteristics of US adults by lifetime use of derived cannabinoid products (*n* = 1523)

Characteristic	Unweighted *n*	Overall Sample	CBD Ever Use % (95% CI)	Delta-8-THC Ever Use % (95% CI)	CBN Ever Use % (95% CI)	CBG Ever Use % (95% CI)	HHC Ever Use % (95% CI)
**Ever Use**			35.2 (32.7–37.9)	7.7 (6.5–9.1)	4.5 (3.7–5.6)	1.3 (0.9–1.9)	1.5 (1.0–2.1)
**Sex**							
Male	747	48.6 (45.4–51.8)	33.6 (30.1–37.2)	8.5 (6.9–10.4)	5.5 (4.2–7.2)	1.7 (1.0–2.8.0.8)	2.1 (1.3–3.3)
Female	776	51.4 (48.2–54.6)	36.8 (33.1–40.6)	6.9 (5.3–9.1)	3.6 (2.6–5.1)	1.0 (0.6–1.7)	0.9 (0.5–1.6)
**Age**							
18–29	227	19.5 (16.8–22.6)	37.3 (30.6–44.6)	9.7 (6.7–13.9)	4.7 (2.8–8.0.8.0)	1.7 (0.7–3.9)	1.5 (0.6–3.8)
30–44	382	25.7 (23.0–28.7.0.7)	39.9 (34.5–45.6)	12.1 (9.1–15.9)	5.0 (3.5–7.0.5.0)	1.6 (0.8–3.0.8.0)	2.6 (1.4–4.7)
45–59	377	23.5 (21.0–26.3.0.3)	33.7 (28.9–38.9)	5.8 (4.2–7.9)	4.3 (2.7–6.8)	1.5 (0.7–3.5)	0.9 (0.4–2.0.4.0)
60+	537	31.2 (28.4–34.0)	31.2 (27.5–35.2)	4.2 (2.9–6.1)	4.2 (2.8–6.3)	0.7 (0.3–1.5)	0.9 (0.4–1.9)
**Race/ethnicity**							
White, Non-Hispanic	1,082	61.8 (58.4–65.1)	38.8 (35.7–42.0)	8.6 (7.1–10.2)	4.4 (3.4–5.7)	1.1 (0.7–1.8)	1.2 (0.7–2.1)
Black, Non-Hispanic	148	12.1 (10.0–14.5.0.5)	32.3 (25.2–40.3)	7.1 (4.4–11.3)	2.3 (1.0–5.2.0.2)	1.1 (0.4–3.6)	1.6 (0.6–4.3)
Other/2 + Races, Non-Hispanic	110	9.1 (7.1–11.5)	24.4 (17.3–31.2)	5.9 (1.9–16.7)	5.1 (2.5–9.9)	1.1 (0.1–7.1)	1.6 (0.4–6.6)
Hispanic	183	17.0 (14.4–20.1)	30.2 (23.9–37.4)	5.8 (3.5–9.6)	6.3 (3.8–10.4)	2.3 (1.0–5.2.0.2)	2.0 (1.0–4.3.0.3)
**Education**							
No high school diploma or GED	92	9.0 (6.9–11.5)	32.9 (23.5–43.9)	9.0 (5.2–14.9)	3.6 (1.6–7.9)	1.8 (0.7–5.1)	2.8 (1.1–6.9)
High school graduate	396	28.8 (25.9–31.9)	34.2 (29.4–39.4)	8.3 (5.8–11.7)	6.6 (4.6–9.4)	1.5 (0.8–3.0.8.0)	1.0 (0.4–2.4)
Some college/Associate’s degree	433	26.6 (24.0–29.4.0.4)	39.8 (34.8–45.0)	8.2 (6.2–10.6)	4.0 (2.6–5.9)	1.9 (1.0–3.7.0.7)	2.6 (1.4–4.6)
Bachelor’s degree or higher	602	35.6 (32.7–38.7)	33.2 (29.5–37.2)	6.5 (4.9–8.7)	3.5 (2.3–5.2)	0.6 (0.3–1.3)	0.6 (0.3–1.4)
**Cannabis Use**							
Yes	954	46.6 (43.5–49.8)	59.5 (55.1–63.8)	15.8 (13.4–18.5)	8.8 (7.1–10.8)	2.5 (1.7–3.7)	2.8 (1.9–4.0.9.0)
No	514	49.3 (46.1–52.5)	12.6 (10.6–14.8)	0.6 (0.2–1.9)	0.8 (0.3–2.2)	0.3 (0.1–1.5)	0.3 (0.1–1.8)
**Other Drug Use**							
Yes	569	28.5 (25.9–31.3)	55.7 (50.1–61.2)	17.0 (14.0–20.5.0.5)	9.7 (7.4–12.5)	3.4 (2.1–5.3)	3.7 (2.3–5.8)
No	954	71.5 (68.7–74.1)	27.1 (24.4–29.8)	4.0 (2.9–5.4)	2.5 (1.7–3.6)	0.5 (0.3–1.0.3.0)	0.6 (0.3–1.1)
**Physical Health**							
Excellent/Very Good	656	44.9 (41.7–48.1)	29.9 (26.5–33.4)	6.5 (5.0–8.4.0.4)	4.2 (3.0–5.8.0.8)	1.1 (0.6–2.0.6.0)	1.3 (0.8–2.3)
Good	530	34.9 (31.9–38.0)	36.4 (32.0–41.1.0.1)	7.4 (5.7–9.6)	4.3 (2.9–6.3)	1.7 (0.9–3.2)	1.3 (0.6–3.0.6.0)
Fair/Poor	319	19.2 (16.8–21.8)	44.5 (37.9–51.3)	11.0 (7.6–15.7)	5.8 (3.7–9.0.7.0)	1.0 (0.4–2.3)	2.1 (1.1–3.8)
**Mental Health**							
Excellent/Very Good	717	48.2 (45.0–51.4.0.4)	29.4 (26.2–32.8)	4.7 (3.5–6.2)	4.2 (3.1–5.8)	0.9 (0.4–1.9)	1.3 (0.8–2.2)
Good	437	29.2 (26.3–32.2)	34.1 (29.5–39)	7.0 (5.3–9.1)	4.7 (3.2–7.0.2.0)	1.5 (0.8–2.8)	1.8 (0.9–3.8)
Fair/Poor	352	21.5 (19.0–24.3.0.3)	49.2 (42.3–56)	15.6 (11.7–20.5)	5.2 (3.4–8.0.4.0)	2.1 (1.1–3.8)	1.4 (0.7–2.8)
**Quality of Life**							
Excellent/Very Good	812	55.5 (52.2–58.6)	28.9 (26.0–32.0)	5.4 (4.1–7.0.1.0)	4.0 (3.0–5.5.0.5)	1.1 (0.6–1.9)	1.1 (0.6–1.9)
Good	471	29.4 (26.5–32.4)	41.4 (36.2–46.8)	8.5 (6.6–10.9)	4.5 (3.1–6.6)	1.1 (0.6–2.1)	1.2 (0.5–3.0.5.0)
Fair/Poor	224	14.2 (12.1–16.6)	46.1 (38.1–54.2)	14.6 (10.0–20.9.0.9)	6.4 (3.8–10.6)	3.0 (1.5–5.9)	3.2 (1.6–6.2)

*CBD* Cannabidiol, *delta-8-THC* Delta-8-tetrahydrocannabinol, *CBN* Cannabinol, *CBG* Cannabigerol, *HHC* Hexahydrocannabinol, *CI* Confidence interval, *GED* General Educational Development

Other drug use includes stimulant, sedative, and tranquilizer misuse, and cocaine, non-prescription stimulant, psychedelic, empathogen, dissociative substance, and other drug use

The patterns of cannabinoid use also varied across demographic subcategories. Lifetime use of all cannabinoids was higher among adults who have used cannabis or other drugs, as well as those rating their quality of life as “fair” or “poor”. After adjusting for other sociodemographic characteristics, there were no differences in derived cannabinoid use by participant race/ethnicity or mental health (Supplemental Table 1). Female sex, younger age, cannabis and other drug use, and good physical health and quality of life were associated with increased odds of CBD use. Younger age, cannabis use, and other drug use were also associated with increased odds of delta-8 THC use. High school education and cannabis and other drug use were associated with increased odds of CBN use.

**Fig. 1:**
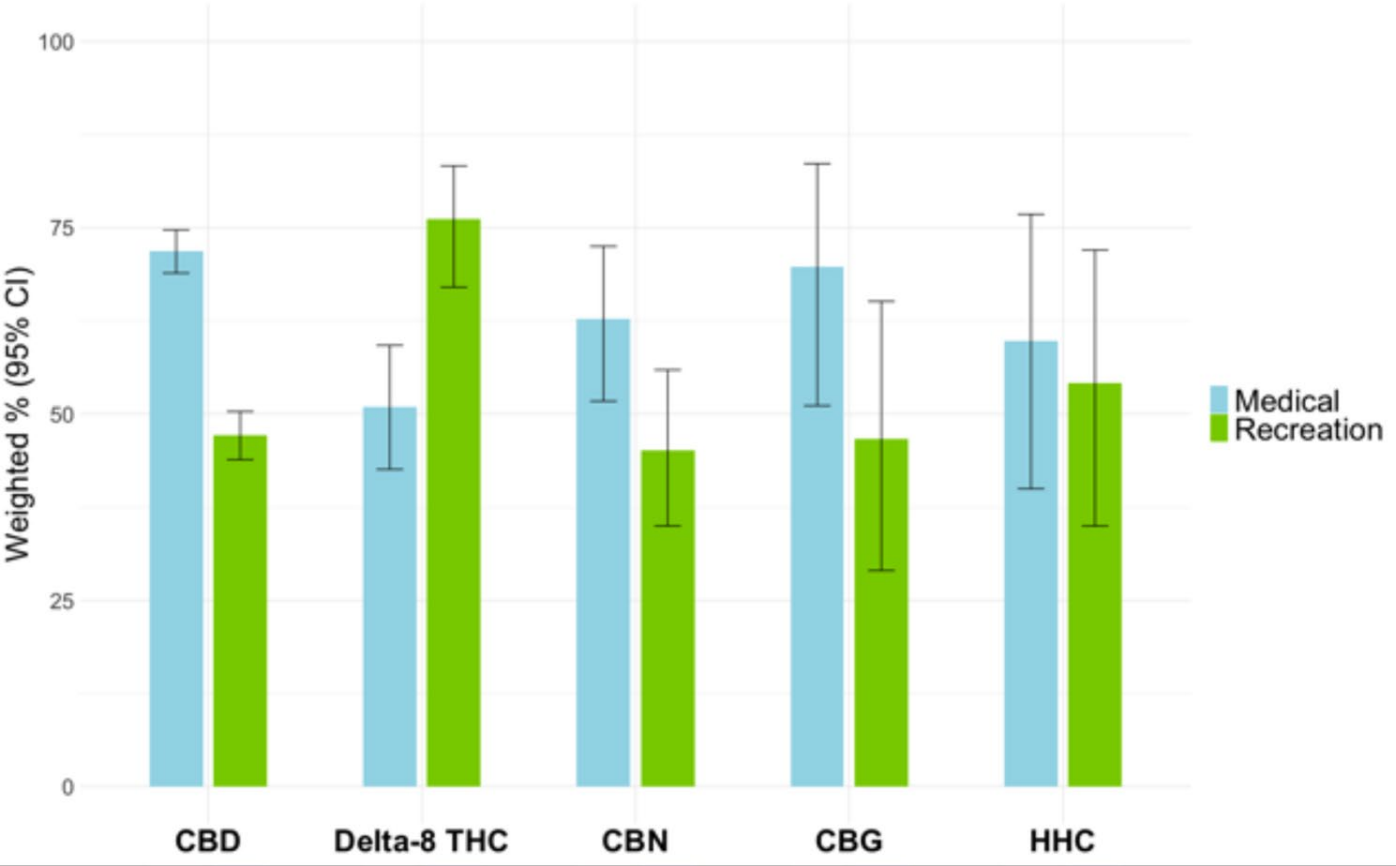
Motivations for cannabinoid product use among a national sample of US adults (n=1523)" and the footnote is "CBD =cannabidiol; delta-8-THC = delta-8-tetrahydrocannabinol; CBN = cannabinol; CBG = cannabigerol; HHC = hexahydrocannabinol; CI= confidence interval

As shown in Table 2, more US adults used CBD for medical purposes only (48.0%, 95% CI 44.8–51.2) than recreational purposes only (23.2%, 95% CI 20.5–26.0) or both (23.9%, 95% CI 21.3–26.8). However, more adults used delta-8 THC for recreational purposes only (45.9% 95% CI 37.8–54.3) than for medical purposes only (20.7%; 95% CI 13.9–29.7) or both (30.2%, 95% CI 23.7–37.6). These differences remained when those who used the products for both reasons were included in both the medical and recreational categories (Fig. 1). More US adults used CBD for medical purposes (71.9%, 95% CI 68.9–74.7) than recreational purposes (47.1%, 95% CI 43.9–50.3). Conversely, more adults used delta-8 THC for recreation (76.1% 95% CI 67.0–83.3.0.3) than for medical reasons (50.9; 95% CI 42.6–59.2). Although more people reported using CBN, CBG, and HHC for medical reasons than recreation, the proportions were unstable due to small sample sizes (Fig. 1).

**Table 2:** Motivations for cannabinoid product use among a National sample of US adults (*n* = 1523)

	Ever Use Unweighted *n*	Reason for Use weighted % (95% CI)		
Cannabinoid		Medical	Recreation	Both
Cannabidiol (CBD)	1,008	48.0 (44.8–51.2)	23.2 (20.5–26.0)	23.9 (21.3–26.8)
Delta-8-THC	191	20.7 (13.9–29.7)	45.9 (37.8–54.3)	30.2 (23.7–37.6)
Cannabinol (CBN)	112	44.9 (34.5–55.8)	27.5 (19.0–38.0)	17.7 (11.6–26.1)
Cannabigerol (CBG)†	31	42.9 (25.1–62.8)	19.7 (9.3–37.0)	26.9 (14.3–44.7)
Hexahydrocannabinol (HHC)†	33	36.3 (20.5–55.7)	30.6 (16.9–48.9)	23.5 (12.3–40.1)

*CBD* Cannabidiol, *delta-8-THC* delta-8-tetrahydrocannabinol, *CBN* Cannabinol, *CBG* Cannabigerol, *HHC* Hexahydrocannabinol, *CI* Confidence interval

†Estimates are unstable as a result of small sample size

Categories do not sum to 100 because participants were allowed to select both medical and recreation use. Estimates for CBG and HHC are unstable as a result of small sample size

Psychiatric disorders were the most common SOC of medical reasons for using delta-8 THC 48.6% (95%CI 41.3–55.9), CBN 36.8% (95%CI 26.3–48.8), and HHC 46.8% (95%CI 23.7–71.5) while musculoskeletal and connective tissue disorders 35.5% (95%CI 32.3–38.8) were the most common SOC for using CBD and general disorders and administration site conditions 39.5% (95%CI 16.2–68.8) were the most common SOC for using CBG (Table 3). General disorders and administration site conditions were the second most frequently mentioned SOC for delta-8 THC 20.0% (95%CI 14.1–27.5), CBN 20.3% (95%CI 12.0–32.4), and HHC 16.5% (95%CI 5.3–41.1) while psychiatric disorders were the second most prevalent SOC for using CBD 33.2% (95%CI 30.3–36.3) and CBG 35.5% (95%CI 15.6–62.2).

**Table 3:** Medical reasons for use among cannabinoid product users by MedDRA system organ class (*n* = 704)

	CBD	Delta-8-THC	CBN	CBG†	HHC†
**MedDRA System Organ Class (SOC)**	weighted % (95% CI)	weighted % (95% CI)	weighted % (95% CI)	weighted % (95% CI)	weighted % (95% CI)
Musculoskeletal and connective tissue disorders	35.5 (32.3–38.8)	15.3 (9.8–23.1)	18.8 (11.0–30.0)	14.6 (4.1–40.8)	15.2 (3.4–47.6)
Psychiatric disorders	33.2 (30.3–36.3)	48.6 (41.3–55.9)	36.8 (26.3–48.8)	35.5 (15.6–62.2)	46.8 (23.7–71.5)
General disorders and administration site conditions	17.1 (15.1–19.3)	20.0 (14.1–27.5)	20.3 (12.0–32.4)	39.5 (16.2–68.8)	16.5 (5.3–41.1)
Nervous system disorders	6.7 (5.4–8.3)	7.8 (4.7–12.9)	6.5 (2.9–14.1)	4.5 (0.9–19.6)	10.3 (1.9–40.7)
Injury, poisoning and procedural complications	1.5 (0.9–2.6)	1.8 (0.3–10.1)	5.1 (1.7–14.6)	-	-
Gastrointestinal disorders	1.3 (0.8–2.2)	3.2 (1.5–6.6)	-	-	-
Skin and subcutaneous tissue disorders	0.8 (0.4–1.6)	-	-	-	-
Surgical and medical procedures	0.5 (0.2–1.2)	0.8 (0.2–3.4)	4.7 (1.6–13.2)	3.3 (0.4–23.4)	11.1 (3.2–32.3)
Metabolism and nutrition disorders	0.5 (0.2–1.0.2.0)	-	0.8 (0.1–5.6)	2.5 (0.3–19.9)	-
Infections and infestations	0.4 (0.2–0.9)	-	-	-	-
Reproductive system and breast disorders	0.4 (0.1–1.1)	-	-	-	-
Respiratory, thoracic and mediastinal disorders	0.4 (0.1–1.1)	0.6 (0.1–4.1)	-	-	-
Immune system disorders	0.3 (0.1–1.1)	-	-	-	-
Investigations	0.3 (0.1–0.8)	-	-	-	-
Neoplasms benign, malignant and unspecified (incl cysts and polyps)	0.2 (0.1–0.6)	-	4.9 (0.7–27.5)	-	-
Hepatobiliary disorders	0.2 (0–1.1.1)	-	-	-	-
Eye disorders	0.2 (0–0.7.7)	1.0 (0.3–4.2)	-	-	-
Congenital, familial and genetic disorders	0.2 (0–0.7.7)	-	2.0 (0.3–12.6)	-	-
Social circumstances	0.1 (0–0.6.6)	0.9 (0.1–5.2)	-	-	-
Blood and lymphatic system disorders	0.1 (0–0.5.5)	-	-	-	-
Vascular disorders	0.1 (0–0.4.4)	-	-	-	-
Renal and urinary disorders	0.1 (0–0.4.4)	-	-	-	-
Cardiac disorders	0 (0–0.2.2)	-	-	-	-

*CBD* Cannabidiol, *delta-8-THC* delta-8-tetrahydrocannabinol, *CBN* Cannabinol, *CBG* Cannabigerol, *HHC* Hexahydrocannabinol, *CI* Confidence interval

†Estimates are unstable as a result of small sample size

As shown in Table 4, the most frequently cited PTs for CBD use were anxiety (14.7%, 95% CI 13.0–16.6), pain (13.1%, 95% CI 11.5–15.0), and arthralgia (11.2%, 95% CI 9.5–13.2). Anxiety (18.6%, 95% CI 13.3–25.3), pain (15.2%, 95% CI 11.1–20.5), and insomnia (10.7%, 95% CI 7.4–15.3) were the most frequently cited preferred terms for delta-8 THC use. Insomnia (15.4%, 95% CI 9.6–23.9), pain (11.1%, 95% CI 6.4–18.7) and anxiety (10.9%, 95% CI 6.0–19.0) were the most frequently cited preferred terms for CBN use. Interestingly, the most frequently cited preferred term category for CBG use could not be determined, coded as “unevaluable event”, followed by anxiety (11.1%, 95% CI 6.4–18.7) and pain (11.1%, 95% CI 6.4–18.7). The most commonly cited preferred terms for HHC use were anxiety (12.0%, 95% CI 3.5–33.7), insomnia (11.3%, 95% CI 2.9–35.3) and pain (10.1%, 95% CI 1.9–40.0). The full list of PTs is shown in Supplemental Table 2.

**Table 4:** Top five medical reasons for use among cannabinoid product users by MedDRA Preferred Term (n=704)

	CBD	Delta-8-THC	CBN	CBG†	HHC†
**MedDRA Preferred Term (PT)**	weighted % (95% CI)	weighted % (95% CI)	weighted % (95% CI)	weighted % (95% CI)	weighted % (95% CI)
Anxiety	14.7 (13.0–16.6)	18.6 (13.3–25.3)	10.9 (6.0–19.0)	17.3 (5.8–41.8)	12.0 (3.5–33.7)
Pain	13.1 (11.5–15.0)	15.2 (11.1–20.5)	11.1 (6.4–18.7)	14.2 (3.7–41.7)	10.1 (1.9–40.0)
Arthralgia	11.2 (9.5–13.2)	-	6.1 (2.9–12.5)	-	4.8 (0.6–30.2)
Insomnia	9.5 (8.1–11.2)	10.7 (7.4–15.3)	15.4 (9.6–23.9)	-	11.3 (2.9–35.3)
Arthritis	6.9 (5.6–8.5)	-	4.4 (1.9–10.0)	-	-
Back pain	-	4.7 (2.3–9.4)	-	-	-
Depression	-	6.0 (3.3–10.4)	-	-	7.6 (1.6–28.8)
Osteoarthritis	-	-	-	4.0 (0.8–18.1)	-
Sleep disorder	-	-	-	5.8 (1.2–23.3)	-
Unevaluable event	-	-	-	25.4 (5.6–66.3)	-

*CBD* Cannabidiol, *delta-8-THC* delta-8-tetrahydrocannabinol, *CBN* Cannabinol, *CBG* Cannabigerol, *HHC* Hexahydrocannabinol, *CI* Confidence interval

†Estimates are unstable as a result of small sample size

Note: Only top 5 PTs are presented for each cannabinoid product, for the full list of PTs, please see Supplemental Table 2

## Discussion

This nationally representative survey provides the first comprehensive assessment of lifetime use and reasons for using various derived cannabinoid products among US adults. Our findings reveal heterogeneity in the prevalence and motivations for cannabinoid use. Use of cannabinoid products is appreciable, with over a third of adults (35.2%) reporting lifetime CBD use, followed by 7.7% adults using delta-8 THC, 4.5% using CBN, 1.3% using CBG, and 1.5% using HHC. Notably, while CBD, CBN, CBG, and HHC were predominantly used for medical purposes, delta-8 THC was more commonly used for recreational purposes. Anxiety, pain, insomnia, and arthralgia (i.e., joint pain) emerged as the primary medical reasons for using the various cannabinoid products assessed. These results underscore the complexity of cannabinoid product use and highlight need for additional research to examine the differences in effects and user profiles of these different products.

Our results are consistent with previous research on cannabinoid product use. We found that lifetime use of CBD among US adults was 35.2%, which was slightly higher than past-year use of CBD (21.1%) estimated by another national survey of US adults [20]. However, our findings showed a lower prevalence of lifetime delta-8 THC, CBN, and CBG use compared to their estimates of past-year use, which may be due to differences in the legal status of cannabis in participants’ states of residence between the two studies. Consistent with findings from an online survey of adults who reported past year delta-8 THC use [32], we found that most adults used delta-8 THC primarily for recreational purposes, similar to delta-9 THC. To our knowledge, our study was the first to estimate the prevalence of HHC among US adults. Although the prevalence of HHC use among our study sample was low (1.5%), it is still concerning due to recent cases of HHC-induced psychotic illness, particularly from legally purchased HHC products [21]. Furthermore, a survey of 106 individuals who reported past six-month HHC use found that nearly 17% experienced adverse effects [33].

Overall, younger, female adults who have worse physical health and quality of life were more likely to use CBD, perhaps because it is often advertised as having benefits for “wellness”. On the other hand, younger adults who use cannabis and illicit substances were more likely to use delta-8 THC, which may be because it is primarily used for its euphoric effects. None of the previous surveys of cannabinoid product users examined mental health, physical health, or quality of life correlates [12, 13, 20, 34].

Similar to online surveys of cannabinoid product users, we found that most individuals who use CBD, delta-8 THC, and CBN for medical reasons use these products for pain and anxiety [10, 13, 14, 35]. These medical applications may result from cannabinoid product manufacturers’ health claims about the therapeutic effects of their products for a variety of medical conditions including pain, arthritis, sleep disorders, and anxiety [16]. For example, an analysis of over 2 million Twitter posts showed that pain, anxiety disorders, sleep disorders, and stress were the four main therapeutic claims for CBD [36]. We found that insomnia was the most common medical reason for CBN use, which is not surprising given that many CBN manufacturers have been shown to make claims about the sleep-promoting effects of CBN [18]. Overall, our results show that US adults are using cannabinoid products for many different medical conditions, possibly as a result of manufacturers making health claims about these products, even though there is a lack of evidence to support these claims. Until there is sufficient scientific evidence that examines the effect of these various cannabinoids on human health, more regulation is needed in regard to the claims manufacturers are allowed to make about cannabinoid products.

The current study has some limitations. The survey was cross-sectional in nature, which does not allow us to measure changes in cannabinoid product use and reasons for use over time. Furthermore, the characteristics of cannabinoid product users and reasons for use were measured in adults, and therefore may not be representative of younger populations. Reason for use was based on self-report using a survey item that mirrors that used in other nationally representative surveys [37], but has the potential to introduce subjectivity in how individuals classify their use. For example, relaxation can be perceived as either medical or recreational. To mitigate this, our survey included a separate category for those that used cannabinoid products for both medical and recreational reasons. However, we did not assess specific reasons for recreational use. Future research is needed to examine how differences in recreational reasons may influence the use of different cannabinoid products. Lastly, we encountered sample size limitations that prevented us from examining individuals who use cannabinoid products more frequently, such as past-year or past-month use. Given that reasons for cannabinoid product use may differ as a function of frequency [38], additional research is needed to examine how motivations for use differ among more frequent users.

## Conclusions

A considerable proportion of US adults have ever used cannabinoid products, especially CBD and delta-8 THC. Most adults reported using CBD, CBN, CBG, and HHC for medical reasons, but delta-8 THC for recreation. Pain, anxiety, insomnia and arthralgia were common medical reasons for use across the different cannabinoids assessed. These differences underscore the complexity of the derived cannabinoid product landscape and the diverse motivations driving their use.

## Supplementary Information

Supplementary Material 1

## Data Availability

The data underlying this article will be shared on reasonable request to the corresponding author.

## Abbreviations

CBD: cannabidiol
CNG: cannabigerol
CBN: cannabinol
FDA: Food and Drug Administration
HHC: hexahydrocannabinol
MDMA: 3,4-Methyl​enedioxy​methamphetamine
MedDRA: Medical Dictionary for Regulatory Activities
MedDRA LLT: MedDRA Lowest Level Term
MedDRA PT: MedDRA Preferred Term
MedDRA SOC: MedDRA System Organ Class
THC: tetrahydrocannabinol
