# Supplementary material for: Prevalence and reasons for using cannabidiol, delta-8 tetrahydrocannabinol, cannabinol, cannabigerol, and hexahydrocannabinol among US adults
Source: J Cannabis Res. 2025 Dec 9;7:100. doi: 10.1186/s42238-025-00359-8 (PMC12690965; doi:10.1186/s42238-025-00359-8)
Supplement: Supplementary file 1 — Supplementary Material 1 [file 42238_2025_359_MOESM1_ESM.docx]

**Supplemental Table 1:** Demographic and behavioral characteristics associated with lifetime cannabinoid product use among a national sample of US adults (n=1498)

| **Characteristic** | **CBD Ever Use aOR (95% CI)†** | **Delta-8-THC Ever Use aOR (95% CI)†** | **CBN Ever Use aOR (95% CI)†** | **CBG Ever Use aOR (95% CI)†*** | **HHC Ever Use aOR (95% CI)†*** |
| --- | --- | --- | --- | --- | --- |
| **Sex** |  |  |  |  |  |
| Male | Ref | Ref | Ref | Ref | Ref |
| Female | **1.36 (1.02-1.81)** | 0.83 (0.57-1.21) | 0.76 (0.47-1.22) | 0.78 (0.38-1.63) | 0.50 (0.21-1.18) |
| **Age** |  |  |  |  |  |
| 18-29 | Ref | Ref | Ref | Ref | Ref |
| 30-44 | **0.61 (0.38-0.97)** | 0.80 (0.46-1.39) | 0.75 (0.37-1.51) | 0.76 (0.25-2.34) | 1.15 (0.40-3.32) |
| 45-59 | **0.57 (0.36-0.91)** | **0.40 (0.23-0.71)** | 0.71 (0.33-1.52) | 0.66 (0.21-2.06) | 0.30 (0.08-1.18) |
| 60+ | **0.48 (0.31-0.75)** | **0.32 (0.18-0.59)** | 0.71 (0.33-1.49) | 0.40 (0.11-1.39) | 0.36 (0.11-1.18) |
| **Race/ethnicity** |  |  |  |  |  |
| White, Non-Hispanic | Ref | Ref | Ref | Ref | Ref |
| Black, Non-Hispanic | 0.88 (0.54-1.42) | 1.05 (0.57-1.95) | 0.66 (0.27-1.62) | 1.37 (0.36-5.17) | 1.65 (0.47-5.81) |
| Other/2+ Races, Non-Hispanic | 0.59 (0.34-1.03) | 0.87 (0.26-2.84) | 1.74 (0.79-3.84) | 1.11 (0.24-5.09) | 1.92 (0.47-7.85) |
| Hispanic | 0.85 (0.53-1.36) | 0.66 (0.32-1.39) | 1.75 (0.84-3.63) | 2.47 (0.97-6.31) | 1.69 (0.58-4.94) |
| **Education** |  |  |  |  |  |
| No high school diploma/ GED | 0.67 (0.35-1.29) | 1.05 (0.48-2.30) | 1.02 (0.33-3.12) | 1.73 (0.32-9.17) | **5.29 (1.32-21.18)** |
| High school graduate | 0.96 (0.66-1.41) | 1.45 (0.77-2.75) | **2.37 (1.23-4.55)** | 2.31 (0.66-8.07) | 1.56 (0.48-5.08) |
| Some college/ Associate's degree | 1.34 (0.95-1.89) | 1.46 (0.87-2.45) | 1.21 (0.62-2.35) | **3.17 (1.06-9.48)** | **4.68 (1.64-13.38)** |
| Bachelor's degree or higher | Ref | Ref | Ref | Ref | Ref |
| **Cannabis Use** |  |  |  |  |  |
| Yes | **9.11 (6.70-12.39)** | **20.81 (6.81-63.56)** | **10.61 (3.62-31.11)** | 5.48 (0.79-37.92) | 5.47 (0.56-53.64) |
| No | Ref | Ref | Ref | Ref | Ref |
| **Other Drug Use** |  |  |  |  |  |
| Yes | **1.55 (1.13-2.14)** | **2.14 (1.36-3.36)** | **2.00 (1.19-3.37)** | **3.55 (1.07-11.77)** | **3.49 (1.04-11.71)** |
| No | Ref | Ref | Ref | Ref | Ref |
| **Physical Health** |  |  |  |  |  |
| Excellent/ Very Good | Ref | Ref | Ref | Ref | Ref |
| Good | **1.42 (1.00-2.01)** | 0.89 (0.51-1.54) | 0.97 (0.50-1.86) | 1.19 (0.36-3.96) | 0.77 (0.30-1.94) |
| Fair/ Poor | 1.21 (0.76-1.92) | 0.64 (0.33-1.25) | 0.94 (0.38-2.33) | 0.18 (0.03-1.14) | 0.51 (0.10-2.55) |
| **Mental Health** |  |  |  |  |  |
| Excellent/ Very Good | Ref | Ref | Ref | Ref | Ref |
| Good | 0.74 (0.49-1.10) | 1.32 (0.72-2.42) | 0.95 (0.48-1.87) | 1.51 (0.44-5.18) | 1.06 (0.43-2.61) |
| Fair/ Poor | 1.04 (0.62-1.72) | 2.03 (0.92-4.46) | 0.51 (0.24-1.08) | 1.29 (0.31-5.32) | 0.20 (0.05-0.81) |
| **Quality of Life** |  |  |  |  |  |
| Excellent/ Very Good | Ref | Ref | Ref | Ref | Ref |
| Good | **1.56 (1.05-2.32)** | 1.00 (0.55-1.80) | 0.92 (0.45-1.91) | 0.62 (0.17-2.25) | 1.03 (0.40-2.62) |
| Fair/ Poor | 1.57 (0.87-2.84) | 1.43 (0.67-3.07) | 1.32 (0.47-3.70) | 2.91 (0.72-11.69) | **5.22 (1.15-23.66)** |

CBD = cannabidiol; CI = confidence interval; delta-8-THC = delta-8-tetrahydrocannabinol; CBN = cannabinol; Ref= reference group.

Other drug use includes stimulant, sedative, and tranquilizer misuse, and cocaine, non-prescription stimulant, psychedelic, empathogen, dissociative substance, and other drug use.

† adjusts for all listed variables

*Estimates are unstable as a result of small sample size

**Supplemental Table 2:** Motivations for cannabinoid product use among a national sample of US adults (n=1523)

|  | **Ever Use  Unweighted n** | **Reason for Use** weighted % (95% CI) | | |
| --- | --- | --- | --- | --- |
| **Cannabinoid** |  | **Medical** | **Recreation** | **Both** |
| Cannabidiol (CBD) | 1,008 | 48.0 (44.8-51.2) | 23.2 (20.5-26.0) | 23.9 (21.3-26.8) |
| Delta-8-THC | 191 | 20.7 (13.9-29.7) | 45.9 (37.8-54.3) | 30.2 (23.7-37.6) |
| Cannabinol (CBN) | 112 | 44.9 (34.5-55.8) | 27.5 (19.0-38.0) | 17.7 (11.6-26.1) |
| Cannabigerol (CBG)† | 31 | 42.9 (25.1-62.8) | 19.7 (9.3-37.0) | 26.9 (14.3-44.7) |
| Hexahydrocannabinol (HHC)† | 33 | 36.3 (20.5-55.7) | 30.6 (16.9-48.9) | 23.5 (12.3-40.1) |

CBD = cannabidiol; delta-8-THC = delta-8-tetrahydrocannabinol; CBN = cannabinol; CBG = cannabigerol; HHC = hexahydrocannabinol; CI = confidence interval

†Estimates are unstable as a result of small sample size

**Supplemental Table 3:** Medical reasons for use among cannabinoid product users by MedDRA Preferred Term (n=704)

|  | **CBD** | **Delta-8-THC** | **CBN** | **CBG†** | **HHC†** |
| --- | --- | --- | --- | --- | --- |
| **MedDRA Preferred Term (PT)** | weighted % (95% CI) | weighted % (95% CI) | weighted % (95% CI) | weighted % (95% CI) | weighted % (95% CI) |
| Anxiety | 14.7 (13.0-16.6) | 18.6 (13.3-25.3) | 10.9 (6.0-19.0) | 17.3 (5.8-41.8) | 10.9 (6.0-19.0) |
| Pain | 13.1 (11.5-15.0) | 15.2 (11.1-20.5) | 11.1 (6.4-18.7) | 14.2 (3.7-41.7) | 11.1 (6.4-18.7) |
| Arthralgia | 11.2 (9.5-13.2) | 3.8 (1.8-7.5) | 6.1 (2.9-12.5) | 3.3 (0.4-23.4) | 6.1 (2.9-12.5) |
| Insomnia | 9.5 (8.1-11.2) | 10.7 (7.4-15.3) | 15.4 (9.6-23.9) | 2.9 (0.3-23.8) | 15.4 (9.6-23.9) |
| Back pain | 7.1 (5.8-8.6) | 4.7 (2.3-9.4) | 3.3 (1.1-9.0) | - | 3.3 (1.1-9.0) |
| Arthritis | 6.9 (5.6-8.5) | 2.7 (1.2-6.0) | 4.4 (1.9-10.0) | 3.3 (0.4-23.4) | 4.4 (1.9-10.0) |
| Myalgia | 5.2 (4.0-6.6) | 1.2 (0.4-3.7) | 1.7 (0.5-5.4) | 1.9 (0.2-16.8) | 1.7 (0.5-5.4) |
| Depression | 3.7 (2.8-4.9) | 6.0 (3.3-10.4) | 3.5 (1.4-8.5) | 2.9 (0.3-21.1) | 3.5 (1.4-8.5) |
| Headache | 1.8 (1.2-2.7) | 1.9 (0.7-5.3) | 0.6 (0.1-4.2) | - | 0.6 (0.1-4.2) |
| Migraine | 1.6 (1.0-2.4) | 1.6 (0.4-5.4) | - | 2.0 (0.2-16.4) | - |
| Unevaluable event | 1.6 (0.9-3.0) | 2.6 (0.7-9.0) | 5.6 (1.3-20.8) | 25.4 (5.6-66.3) | 5.6 (1.3-20.8) |
| Post-traumatic stress disorder | 1.3 (0.8-2.1) | 1.5 (0.4-5.2) | 1.0 (0.1-6.9) | - | 1.0 (0.1-6.9) |
| Stress | 1.3 (0.8-2.2) | 3.0 (1.3-6.4) | 1.7 (0.4-6.7) | 2.9 (0.3-22.6) | 1.7 (0.4-6.7) |
| Inflammation | 1.1 (0.6-1.8) | - | 2.4 (0.7-7.3) | - | 2.4 (0.7-7.3) |
| Fibromyalgia | 0.9 (0.5-1.7) | - | 1.2 (0.3-4.8) | - | 1.2 (0.3-4.8) |
| Feeling of relaxation | 0.7 (0.4-1.3) | 0.6 (0.1-4.4) | - | - | - |
| Neck pain | 0.6 (0.3-1.2) | - | - | - | - |
| Neuropathy peripheral | 0.6 (0.3-1.1) | 0.4 (0.1-3.1) | 2.3 (0.8-7.1) | - | 2.3 (0.8-7.1) |
| Rheumatoid arthritis | 0.6 (0.3-1.3) | 0.6 (0.1-4.2) | - | - | - |
| Sleep disorder | 0.6 (0.3-1.3) | 0.9 (0.2-3.7) | 1.3 (0.3-5.6) | 5.8 (1.2-23.3) | 1.3 (0.3-5.6) |
| Osteoarthritis | 0.5 (0.3-1.1) | 0.3 (0.0-2.5) | - | 4.0 (0.8-18.1) | - |
| Seizure | 0.5 (0.2-1.2) | 0.7 (0.1-4.8) | 1.0 (0.1-7.1) | - | 1.0 (0.1-7.1) |
| Attention deficit hyperactivity disorder | 0.4 (0.1-1.0) | 2.4 (0.8-6.5) | - | - | - |
| Epilepsy | 0.4 (0.2-0.9) | 0.4 (0.1-3.2) | 0.7 (0.1-5.0) | 2.5 (0.3-19.9) | 0.7 (0.1-5.0) |
| Multiple sclerosis | 0.4 (0.1-1.0) | - | - | - | - |
| Neuralgia | 0.4 (0.2-0.9) | - | - | - | - |
| Sciatica | 0.4 (0.2-0.9) | 0.5 (0.1-3.7) | - | - | - |
| Muscle spasms | 0.3 (0.1-0.7) | - | - | - | - |
| Dysmenorrhoea | 0.3 (0.1-0.9) | - | - | - | - |
| Skin disorder | 0.3 (0.1-0.7) | - | - | - | - |
| Joint injury | 0.3 (0.1-1.0) | - | - | - | - |
| Mental disorder | 0.3 (0.1-1.0) | 1.3 (0.3-5.3) | 2.0 (0.3-12.6) | - | 2.0 (0.3-12.6) |
| Parkinson's disease | 0.3 (0.1-0.7) | 0.4 (0.1-2.7) | 1.3 (0.3-5.4) | - | 1.3 (0.3-5.4) |
| Intervertebral disc degeneration | 0.3 (0.1-0.8) | 0.4 (0.1-3.1) | - | 2.0 (0.2-16.4) | - |
| Decreased appetite | 0.2 (0.0-0.7) | - | 0.8 (0.1-5.6) | - | 0.8 (0.1-5.6) |
| Autism spectrum disorder | 0.2 (0.0-0.8) | 0.6 (0.1-4.6) | 1.0 (0.1-7.2) | 3.6 (0.4-27.9) | 1.0 (0.1-7.2) |
| Crohn's disease | 0.2 (0.1-0.7) | 0.8 (0.2-3.5) | - | - | - |
| Diabetes mellitus | 0.2 (0.0-0.6) | - | - | - | - |
| Dyspepsia | 0.2 (0.0-0.8) | - | - | - | - |
| Dry skin | 0.2 (0.1-0.9) | - | - | - | - |
| Gastrointestinal pain | 0.2 (0.1-0.8) | - | - | - | - |
| Glaucoma | 0.2 (0.0-0.7) | 0.5 (0.1-3.8) | - | - | - |
| Pain in extremity | 0.2 (0.0-0.6) | 0.4 (0.1-3.3) | - | - | - |
| Musculoskeletal stiffness | 0.2 (0.1-0.7) | - | - | - | - |
| Nervousness | 0.2 (0.0-0.7) | 0.6 (0.1-4.4) | - | - | - |
| Panic attack | 0.2 (0.0-0.6) | 0.5 (0.1-3.6) | - | - | - |
| Plantar fasciitis | 0.2 (0.0-0.6) | - | - | - | - |
| Psoriasis | 0.2 (0.0-0.6) | - | - | - | - |
| Weight | 0.2 (0.0-0.6) | - | - | - | - |
| General physical health deterioration | 0.2 (0.0-0.7) | - | - | - | - |
| Muscle strain | 0.2 (0.1-0.7) | 0.9 (0.1-5.2) | - | - | - |
| Limb injury | 0.2 (0.0-0.8) | - | - | - | - |
| Alopecia | 0.1 (0.0-0.5) | - | - | - | - |
| Ankle fracture | 0.1 (0.0-0.8) | - | - | - | - |
| Polyarthritis | 0.1 (0.0-0.5) | - | - | - | - |
| Contusion | 0.1 (0.0-0.8) | - | - | - | - |
| Bursitis | 0.1 (0.0-0.9) | - | 1.2 (0.2-8.6) | - | 1.2 (0.2-8.6) |
| Neoplasm malignant | 0.1 (0.0-0.5) | - | 4.9 (0.7-27.5) | - | 4.9 (0.7-27.5) |
| Ex-tobacco user | 0.1 (0.0-0.6) | - | - | - | - |
| Chronic hepatitis | 0.1 (0.0-0.6) | - | - | - | - |
| Cystitis interstitial | 0.1 (0.0-0.4) | - | - | - | - |
| Nasopharyngitis | 0.1 (0.0-0.5) | - | - | - | - |
| Disturbance in attention | 0.1 (0.0-0.6) | 0.5 (0.1-3.7) | - | - | - |
| Constipation | 0.1 (0.0-0.4) | - | - | - | - |
| Chronic obstructive pulmonary disease | 0.1 (0.0-0.5) | - | - | - | - |
| Ehlers-Danlos syndrome | 0.1 (0.0-0.5) | - | - | - | - |
| Epstein-Barr virus infection | 0.1 (0.0-0.5) | - | - | - | - |
| Malaise | 0.1 (0.0-0.7) | - | - | - | - |
| Generalised anxiety disorder | 0.1 (0.0-0.6) | 0.5 (0.1-3.9) | - | - | - |
| Gastrooesophageal reflux disease | 0.1 (0.0-0.6) | 0.5 (0.1-3.6) | - | - | - |
| Gout | 0.1 (0.0-0.5) | - | - | - | - |
| Hip arthroplasty | 0.1 (0.0-0.9) | - | 0.9 (0.1-6.4) | 3.3 (0.4-23.4) | 0.9 (0.1-6.4) |
| HIV infection | 0.1 (0.0-0.5) | - | - | - | - |
| Hypertension | 0.1 (0.0-0.4) | - | - | - | - |
| Injection site pain | 0.1 (0.0-0.9) | - | 1.2 (0.2-8.4) | - | 1.2 (0.2-8.4) |
| Irritable bowel syndrome | 0.1 (0.0-0.5) | - | - | - | - |
| Antibiotic therapy | 0.1 (0.0-0.6) | - | - | - | - |
| Lyme disease | 0.1 (0.0-0.5) | - | - | - | - |
| Euphoric mood | 0.1 (0.0-0.6) | - | - | - | - |
| Mood swings | 0.1 (0.0-0.6) | - | - | - | - |
| Muscle injury | 0.1 (0.0-0.4) | - | - | - | - |
| Nausea | 0.1 (0.0-0.6) | 1.5 (0.4-5.1) | - | - | - |
| Nerve injury | 0.1 (0.0-0.5) | - | - | - | - |
| Osteoporosis | 0.1 (0.0-0.6) | 0.5 (0.1-3.9) | 0.8 (0.1-6.0) | - | 0.8 (0.1-6.0) |
| Psoriatic arthropathy | 0.1 (0.0-0.4) | - | - | - | - |
| Restless legs syndrome | 0.1 (0.0-0.6) | 0.5 (0.1-3.6) | - | - | - |
| Rotator cuff syndrome | 0.1 (0.0-0.6) | - | - | - | - |
| Osteochondrosis | 0.1 (0.0-0.5) | - | - | - | - |
| Schizophrenia | 0.1 (0.0-0.9) | - | - | - | - |
| Sciatic nerve injury | 0.1 (0.0-0.9) | - | 1.2 (0.2-8.6) | - | 1.2 (0.2-8.6) |
| Herpes zoster | 0.1 (0.0-0.4) | - | - | - | - |
| Sickle cell disease | 0.1 (0.0-0.7) | - | - | - | - |
| Sleep apnoea syndrome | 0.1 (0.0-0.8) | - | - | - | - |
| Poor quality sleep | 0.1 (0.0-0.5) | - | - | - | - |
| Abdominal pain upper | 0.1 (0.0-0.7) | - | - | - | - |
| Cerebrovascular accident | 0.1 (0.0-0.5) | 0.4 (0.1-3.0) | - | - | - |
| Surgery | 0.1 (0.0-0.6) | 0.4 (0.1-2.8) | - | - | - |
| Peripheral swelling | 0.1 (0.0-0.6) | - | - | - | - |
| Tendonitis | 0.1 (0.0-0.5) | - | - | - | - |
| Epicondylitis | 0.1 (0.0-0.4) | - | - | - | - |
| Temporomandibular pain and dysfunction syndrome | 0.1 (0.0-0.6) | - | - | - | - |
| Tremor | 0.1 (0.0-0.6) | - | - | - | - |
| Immunodeficiency | 0.1 (0.0-0.6) | - | - | - | - |
| Weight decreased | 0.1 (0.0-0.5) | - | - | - | - |
| Post-traumatic neck syndrome | 0.1 (0.0-0.4) | - | - | - | - |
| Pain of skin | 0.1 (0.0-0.5) | - | - | - | - |
| Muscle tightness | 0.1 (0.0-0.4) | - | - | - | - |
| Wound | 0.1 (0.0-0.5) | - | - | - | - |
| Routine health maintenance | 0.1 (0.0-0.8) | - | - | - | - |
| Procedural pain | 0.1 (0.0-0.5) | - | 3.2 (0.6-14.5) | - | 3.2 (0.6-14.5) |
| Bipolar disorder | 0.1 (0.0-0.9) | 0.6 (0.1-4.4) | - | - | - |
| Autoimmune disorder | 0.1 (0.0-0.6) | - | - | - | - |
| Spondylolisthesis | 0.1 (0.0-0.7) | 0.6 (0.1-4.6) | - | - | - |
| Bone loss | 0.1 (0.0-1.0) | - | - | - | - |
| Mast cell activation syndrome | 0.1 (0.0-0.5) | - | - | - | - |
| Delusion of parasitosis | 0.1 (0.0-0.5) | - | - | - | - |
| Sleep deficit | 0.1 (0.0-0.6) | - | - | - | - |
| Major depression | 0.1 (0.0-0.5) | - | - | - | - |
| Knee arthroplasty | 0.1 (0.0-0.4) | - | 0.8 (0.1-5.6) | - | 0.8 (0.1-5.6) |
| Alcoholism | 0.1 (0.0-0.4) | 0.3 (0.0-2.5) | - | - | - |
| Ankylosing spondylitis | 0.1 (0.0-0.8) | - | - | - | - |
| Asthma | 0.1 (0.0-0.6) | 0.6 (0.1-4.1) | - | - | - |
| Blood pressure measurement | 0.1 (0.0-0.4) | - | - | - | - |
| Hepatic cirrhosis | 0.1 (0.0-0.6) | - | - | - | - |
| Cyst | 0.1 (0.0-0.6) | - | - | - | - |
| Endometriosis | 0.1 (0.0-0.6) | - | - | - | - |
| Hypersensitivity | 0.1 (0.0-1.0) | - | - | - | - |
| Radiculopathy | 0.1 (0.0-0.5) | - | 0.7 (0.1-4.7) | - | 0.7 (0.1-4.7) |
| Chemotherapy | 0.1 (0.0-0.5) | - | 2.5 (0.4-14.9) | - | 2.5 (0.4-14.9) |
| Menopause | 0.1 (0.0-0.5) | - | - | - | - |
| Borderline personality disorder | 0.1 (0.0-0.9) | - | - | - | - |
| Breast cancer | 0.1 (0.0-0.5) | - | - | - | - |
| Chronic hepatitis B | 0.1 (0.0-0.6) | - | - | - | - |
| Joint stiffness | 0.1 (0.0-0.6) | - | - | - | - |
| Toothache | 0.1 (0.0-1.0) | - | - | - | - |
| Thinking abnormal | - | 0.5 (0.1-3.6) | - | - | - |
| Back injury | - | - | 0.7 (0.1-4.7) | - | 0.7 (0.1-4.7) |
| Cachexia | - | - | - | 2.5 (0.3-19.9) | - |
| Cardiomyopathy | 0.0 (0.0-0.2) | - | - | - | - |
| Carpal tunnel syndrome | - | 0.5 (0.1-3.7) | - | - | - |
| Cerebral palsy | - | - | 2.0 (0.3-12.6) | - | 2.0 (0.3-12.6) |
| Colitis | - | 0.5 (0.1-3.3) | - | - | - |
| Compression fracture | 0.0 (0.0-0.2) | - | - | - | - |
| Injury | 0.0 (0.0-0.4) | - | - | - | - |
| Hepatic cancer | 0.0 (0.0-0.2) | - | - | - | - |
| Asthenia | - | 1.6 (0.2-10.5) | - | - | - |
| Lymphoma | 0.0 (0.0-0.2) | - | - | - | - |
| Analgesic therapy | - | 0.4 (0.1-3.2) | - | - | - |
| Vision blurred | - | 0.5 (0.1-3.7) | - | - | - |
| Underweight | 0.0 (0.0-0.3) | - | - | - | - |
| Depressed mood | - | - | - | - | - |
| Ophthalmologic treatment | - | - | - | - | - |
| Anhedonia | - | 0.6 (0.1-4.1) | - | - | - |
| Ligament sprain | - | 0.9 (0.1-5.2) | - | - | - |
| Substance use | - | 0.9 (0.1-5.2) | - | - | - |
| Pain management | - | - | 0.5 (0.1-4.0) | - | 0.5 (0.1-4.0) |

CBD = cannabidiol; CI = confidence interval; delta-8-THC = delta-8-tetrahydrocannabinol; CBN = cannabinol; Ref= reference group.

† Estimates are unstable as a result of small sample size
